# Impact of Physiological and Psychological Stress on Glaucoma Development and Progression: A Narrative Review

**DOI:** 10.3390/medicina61030418

**Published:** 2025-02-27

**Authors:** Lauren J. Isserow, Danielle Harris, Nathan Schanzer, Brent Siesky, Alice Verticchio Vercellin, Keren Wood, Fani Segev, Alon Harris

**Affiliations:** 1 Department of Ophthalmology, Icahn School of Medicine at Mount Sinai, New York, NY 10029, USA; lauren.isserow@rutgers.edu (L.J.I.); nathan.schanzer@mssm.edu (N.S.); keren.woodshalem@mssm.edu (K.W.); 2 Department of Psychology, Reichman University, University Street 8, Herzellia 4610101, Israel; danielle.harris@post.runi.ac.il; 3 Department of Ophthalmology, Samson Assuta Ashdod University Hospital, Ashdod 7747629, Israel

**Keywords:** acute stress, chronic stress, environmental stress, primary open-angle glaucoma, primary angle-closure glaucoma, intraocular pressure, mindfulness, mental health, socioeconomic status

## Abstract

Glaucoma is a leading cause of irreversible blindness worldwide. Presently, elevated intraocular pressure (IOP) is the only approved modifiable risk factor. A consensus of the current literature suggests that both physiological and psychological stress may also impact the lifelong course of glaucoma. Specifically, stress is known to influence sympathetic nervous system activity. An increase in sympathetic nervous system activity may elevate a person’s blood pressure (BP) and IOP, and both are strongly associated with glaucomatous disease. Anxiety and depression have more conflicting evidence in relation to glaucoma. Socioeconomic and environmental stress may worsen adherence to therapy and disease outcomes due to a lack of financial resources and related access to healthcare. Neighborhood quality and environmental conditions, particularly urban environments, have been associated with glaucoma risk factors, higher glaucoma prevalence, and delayed surgical interventions. Racial differences have also been identified, with Black patients being more stressed and likely to present with increased glaucoma severity and faster disease progression than White patients. Mindfulness, meditation, and other forms of psychological relaxation have been shown to reduce IOP and stress biomarkers and result in improved quality of life (QOL). Larger studies in more diverse populations are needed to clarify risk and identify the best therapeutic approaches to reduce stress as a method to improve clinical outcomes and QOL for glaucoma patients.

## 1. Introduction

As a leading cause of irreversible blindness worldwide, glaucoma affects approximately 3.5% of the global population aged 40–80 years old [1]. Currently, the disease impacts over 76 million people worldwide, and is projected to affect up to 112 million persons by 2040 [2]. Risk factors for developing glaucoma include older age, family history, being non-White and having elevated intraocular pressure (IOP) [1,2]. Elevated IOP is the only currently approved modifiable risk factor for the condition. This represents a significant limitation to patient care as all current therapeutic interventions work only to lower IOP, which is not effective for all patients [2]. In addition, glaucoma is a chronic disease; therefore, patients require lifelong treatment following initial diagnosis [1].

Glaucomatous disease is defined by a progressive neuropathy of the optic nerve, death of retinal ganglion cells (RGCs) and thinning of the retinal nerve fiber layer (RNFL) leading to visual field (VF) loss [1,2,3]. Initially, glaucoma may be asymptomatic, with many unaware of initial peripheral vision loss [1]. If left untreated, glaucoma may progress and cause central vision loss and irreversible blindness [1,2]. Glaucoma is frequently subdivided into categories including open-angle glaucoma (OAG) and angle-closure glaucoma (ACG) based on the ability of aqueous humor to drain from the eye. In OAG, the aqueous outflow pathway is patent, although flow is decreased [1]. ACG is characterized by a greater than 270-degree blockage of the iridocorneal angle, functionally blocking aqueous drainage [1]. Glaucoma is treated with medications, laser, or surgery, with all current treatment paradigms focused on lowering IOP [1]. The diagnosis and management of glaucoma are currently hindered by the cost and expertise required to adequately diagnose the disease [4]. Cutting-edge artificial intelligence (AI) methods applied to fundus photography have recently shown to reliably identify glaucoma patients [5,6,7]. AI approaches, therefore, have the potential to improve accessibility to disease diagnostics and access to treatment [5,6,7].

**Figure 1:**
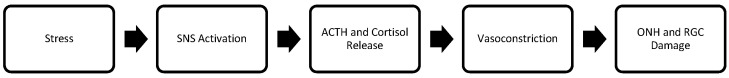
Pathophysiology of stress-induced glaucomatous damage. ACTH: adrenocorticotropic hormone; ONH: optic nerve head; RGC: retinal ganglion cell; SNS: sympathetic nervous system.

“Stress”, a term coined by Hans Seyle in the 1930s, is a common condition of daily life. Acute stress, chronic stress, and socioeconomic stress have all been associated with worse outcomes in chronic illnesses such as cardiovascular disease (CVD), hypertension, end-stage renal disease (ESRD), and type two diabetes mellitus (T2DM) [8,9,10]. The relationship between stress and negative health outcomes, however, is multifaceted. Chronic activation of the sympathetic nervous system, leading to hormone production, such as adrenocorticotropic hormone (ACTH) and cortisol, via the hypothalamus–pituitary axis, is associated with increased incidences of hypertension and cardiovascular disease [11] (Figure 1). The relationship between systemic BP and IOP, however, is complex. In a previous systematic review, Zhao et al. (2014) discussed a widely identified positive relationship between systolic BP, diastolic BP, and IOP [12]. Additionally, evidence suggests that chronic exposure to endogenous stress-related hormones can directly lead to damage of the optic nerve head (ONH) and RGCs through catecholamine-mediated vasoconstriction reducing blood flow [13].

The ability to cope with and manage chronic illness may also be affected by various forms of stress. In 1977, George Engel advocated for the advancement of the biomedical model to the biopsychosocial model to account for medical and psychological factors contributing to disease process [14]. He suggests somatic diseases such as diabetes and mental diseases such as schizophrenia should not be viewed solely from their respective lenses, and should be viewed holistically considering medical, psychological, and cultural influences [14]. Studies of chronic illnesses such as CVD, ESRD, and T2DM suggest that outcomes are a result of a dynamic interplay of psychosocial factors such as age, gender, race, socioeconomic status, environmental conditions, personality, and culture [9,10,15]. Many researchers have proposed a connection between various types of stress and glaucoma as a chronic disease to understand risk factors and potential treatment strategies. This review evaluates evidence of various types of stress, including acute stress, chronic stress, psychological stress (anxiety, depression, etc.), socioeconomic stress, racial stress, environmental stress, and stress reduction techniques and their impact on glaucoma.

## 2. Materials and Methods

A narrative review of the literature discussing stress and glaucoma was conducted from 1 January 1996 to 31 December 2024, using PubMed, Embase, Ovid, Scopus, and reference list cross-matching of related articles. The same key words were used in all search software, with articles screened for relevance, and data were collected and organized using Microsoft Word (Microsoft Corporation, Redmond, WA, USA). Search terms included “stress and glaucoma”, “psychosocial model and glaucoma”, “biopsychosocial model glaucoma”, “acute stress and glaucoma”, “chronic stress and glaucoma”, “allostatic load and glaucoma”, “socioeconomic status and glaucoma”, “poverty and glaucoma”, “racism and glaucoma”, “gender and glaucoma”, “anxiety and glaucoma”, “depression and glaucoma”, “environment and glaucoma”, “discrimination and glaucoma”, “stress reduction and glaucoma”, and “mindfulness and glaucoma.”

**Figure 2:**
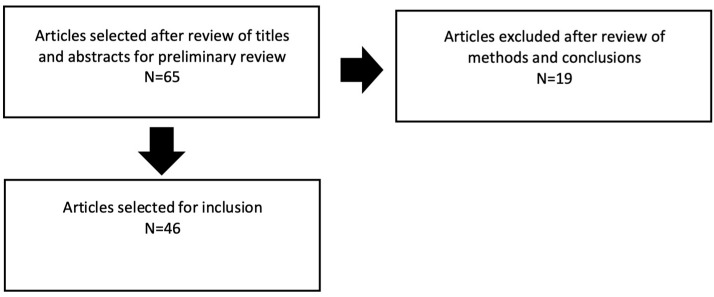
Flowchart of article selection process.

Based on the search criteria, a total of 65 studies were selected for further consideration, with 19 studies then excluded due to non-relevance. In total, 46 studies consisting of retrospective studies, prospective studies, randomized controlled trials, case reports, case–control studies, and cross-sectional prevalence studies were deemed relevant and included in this narrative review (Figure 2).

## 3. Results

The literature review yielded six main types of stress and their relationship to glaucoma divided into the following relevant sections:

(1) Acute mental stress;
(2) Chronic stress;
(3) Mental health stress;
(4) Socioeconomic stress;
(5) Racial stress;
(6) Environmental stress;
(7) Mindfulness and stress reduction.

## 4. Discussion

### 4.1. Acute Stress

Acute stress may increase the risk for the development and progression of glaucoma. Studies have shown that acute stress can cause a significant elevation of BP and IOP via activation of the sympathetic nervous system [16,17,18]. Both elevated IOP and BP have been strongly linked to glaucomatous disease. Among acute stress studies, evidence suggests that fluctuations in the sympathetic nervous system may lead to IOP fluctuations and thus exacerbate glaucomatous disease in adult and pediatric populations [11,16,19,20,21,22]. In addition to IOP, some data have demonstrated a link between stress and RNFL thickness, suggesting a potential impact of chronic stress on retinal tissues [23].

Elevated IOP is a major risk factor for glaucoma. The sympathetic nervous system is intrinsically involved with mediating fluctuations in aqueous humor flow and IOP [16]. Acute mental stressors, such as those conducted in mental stressor tests, significantly impact IOP. Kaluza et al. (1996) investigated the effects of a mental stressor test (MST) on IOP in 23 OAG patients [19]. In this MST, patients were required to complete mathematical operations while listening to gunfire, sirens, and abnormal breathing instructions [19]. A statistically significant increase in IOP was found following the MST, from 17.7 mmHg at baseline to 19.1 mmHg after the MST (*p* < 0.01), associated with an increase in perceived psychological strain (*p* < 0.01) [19]. In another study examining the effects of an MST on IOP, Ferreira et al. (2024) investigated the effects of the Trier Social Stress Test (TSST), where participants are required to make a presentation and conduct a surprise mathematical assessment, while being watched by an interview panel, on IOP in OAG patients [21,24]. Increases in IOP of 3.8 mmHg (*p* < 0.0001) and 4.1 mmHg (*p* < 0.0001) were observed in the right and left eyes, respectively, from baseline to immediately after TSST [21]. This post-TSST increase in IOP was also associated with a 5.9 nmol/L increase in salivary cortisol (*p* = 0.004), a 10.1 mmHg increase in mean arterial pressure (*p* < 0.001), an increase in heart rate (*p* < 0.001), and an increase in State Trait Anxiety Inventory (*p* < 0.001) [21]. While these studies showed an increase in IOP following an acute mental stressor, neither study investigated long-term consequences of these IOP elevations. Future research should investigate the long-term effects of mental stress associated IOP elevation.

University student populations have been identified as having elevated stress and IOP. Abokyi et al. (2024) and Jimenez and Vera (2017) measured the IOP and ocular perfusion pressure (OPP) of 36 healthy participants before and after a stressful examination, and before and after a non-stressful class (control) [16,22]. Following Bayesian analysis, there was a statistically significant increase in IOP under examination conditions compared to the control (BF_01_ < 0.001) [16]. IOP prior to the exam was statistically significantly higher (19.1) than after the exam (17.3) (BF_01_ = 0.078) [16]. Additionally, increases in systolic BP (BF_01_ < 0.001), diastolic BP (BF_01_ < 0.001), and OPP (BF_01_ < 0.001) were found in the examination condition compared to the control [16]. Similarly, Abokyi et al. (2024) previously studied IOP in healthy university students and university students with juvenile-onset open-angle glaucoma (JOAG) at the beginning of the semester (low stress) and at the end of the semester (high stress) [22]. The authors observed a 3.07 mmHg increase in IOP in those with JOAG and a 2.3 mmHg increase in IOP in healthy participants at the end of the semester compared to the beginning of the semester, associated with statistically significant increases in perceived stress (*p* < 0.001). Factorial analysis demonstrated that stress has a greater impact on the IOP of participants with JOAG than healthy participants (*p* < 0.001) [22].

Acute stressful interpersonal situations in susceptible populations may also worsen glaucoma. Gillmann et al. (2019) presented a case study of a 78-year woman with a history of type A personality, pseudoexfoliative glaucoma, a surgical history of Ex-PRESS shunt placement in the left eye, and deep sclerotomy and YAG goniopuncture in the right eye presenting with acute IOP elevation after an acutely stressful personal event [11]. Prior to this episode, her left IOP was between 14 and 19 mmHg and her right IOP was between 16 and 21 mmHg. She maintained constant medical management of latanoprost (Xalatan, Pfizer PFE Switzerland GmbH, Steinhausen, Switzerland) and timolol 0.1% (Timogel, Théa Pharma SA, Schaffhouse, Switzerland), as directed by her physicians prior to this exacerbation. On exam, her right eye IOP was 18 mmHg and her left eye IOP was 48 mmHg with an anterior segment optical coherence tomography-confirmed patent Ex-PRESS shunt. The patient described a period of recent extreme family stress and participation in an intense argument prior to her examination. Following treatment with topical timolol and dorzolamide (Cosopt, Santen, Osaka, Japan), brimonidine (Alphagan, Allergan, Dublin, Ireland), and oral acetazolamide (Diamox, Vifor Pharma, Glattbrugg Switzerland), her IOP normalized to 10 mmHg in the right eye and 16 mmHg in the left eye the following day. Two months after her initial presentation, her IOP remained stable [11]. However, since this is a single case report, additional research in larger populations is needed.

VF defects may be present following MST-induced IOP elevation. Keren et al. (2021) studied IOP and VF in 36 participants with OAG before and after Stroop mental stress testing, a 20 min stress test where participants must identify the color written in discordant color font, under pressure from examiners [20]. They found a statistically significant increase in IOP following the Stroop mental stress test from 15 mmHg to 16 mmHg (*p* < 0.001) in 36 patients. The IOP increase was also associated with a decrease in VF outcomes as measured by the mean deviation (MD) (*p* = 0.008) [20]. However, the authors did not investigate long-term VF defects associated with MST-induced IOP elevations. Therefore, additional research is required to evaluate the long-term effects on visual field outcomes induced by mental stress-related IOP increase.

Although there is established evidence that emotional stress may contribute to elevations in IOP, recent research suggests that emotional stress may be directly related to changes in the RNFL. A study by Lee et al. (2020) suggests that it may be necessary to understand the impact of acute stress-related IOP elevations on RNFL thickness [23]. An analysis of 863 healthy participants’ cortisol and ACTH levels was conducted before and after completing the TSST. There was no statistically significant relationship between cortisol levels and RNFL thickness. The authors found a statistically significant, inverse relationship between pre-TSST ACTH and RNFL thickness globally (*p* = 0.009) and specifically in the inferotemporal (*p* = 0.015), temporal (*p* = 0.046), and superotemporal (*p* = 0.044) retina. However, the effect is small, whereby a 10 pg/nL increase in ACTH corresponds to a 1.2–2.3 μm decrease in baseline RNFL thickness. Lee et al. (2021) also found a positive association between adrenal sensitivity (defined as the ratio of cortisol area under the curve with respect to ground/ACTH area under the curve with respect to ground) and RNFL thickness temporally (*p* = 0.037) and inferotemporally (*p* < 0.001) [23].

Collectivity, these studies describe how acutely stressful situations may increase IOP via the sympathetic nervous system in healthy participants and those with OAG. This acute elevation in IOP may be associated with a potential VF loss. Additionally, there is a small decrease in RNFL thickness in healthy participants associated with higher baseline ACTH, a biochemical marker for elevated stress state. These findings provide evidence for the exacerbation of glaucomatous disease in patients with acute stress exacerbations.

### 4.2. Chronic Stress

Chronic stress and allostatic load (AL) are defined as the physical consequences of exposure to long-term stressful conditions and are strongly associated with various chronic conditions, including hypertension, T2DM, and breast cancer [13]. In addition to the previously stated conditions, it is currently known that glaucoma diagnoses are associated with biochemical evidence of chronic disease and chronic stress [13,24,25]. Additionally, chronic stress related to personality types may also be a risk factor for glaucoma [26]. Moreover, AL is a risk factor for medication noncompliance and, thus, worsening of glaucoma [27].

In terms of assessing AL, GC et al. (2024) investigated the effects of AL on 50 participants with primary OAG (POAG) and 50 healthy controls [13]. Data analysis suggested higher AL for patients with POAG (*p* = 0.0002), with levels above biological cutoffs in POAG patients for glycosylated hemoglobin (HbA1c) (*p* = 0.003), total cholesterol (*p* = 0.037), high-density lipoprotein (*p* = 0.005), and homocysteine (*p* = 0.001). However, although cortisol levels were higher in the POAG group than the control group, this difference was not statistically significant (*p* = 0.24) [13]. While the authors present correlations between elevated biochemical markers and glaucoma, more research is needed to further the understanding of the relationship between these biomarkers and disease risk.

In a retrospective case–control study of glaucoma patients and healthy controls using the National Institute of health All of Us Research Program data, Yoo et al. (2024) found that higher AL is associated with an increased odds ratio (OR) for glaucoma diagnosis (OR = 1.09 per point AL increase) [24]. Specifically, increased AL is associated with POAG diagnosis (OR 1.11; *p* = 0.01) but is not associated with a risk of developing primary ACG (PACG) (*p* = 0.87). Additionally, like Gc et al. (2024), Yoo et al. (2024) found higher HbA1C (*p* < 0.001) and homocysteine levels (*p* = 0.01) in participants with glaucoma compared to controls [13,24]. However, no differences were found in total cholesterol levels (*p* = 0.22) between glaucoma participants and healthy controls [24]. These findings are limited by the far smaller sample size of the glaucoma group (349) compared to the control group (1819) [24]. Additionally, these measurements were not drawn at consistent time intervals; therefore, biases may be present depending on when in the disease process these biomarkers were studied [24].

In terms of chronic comorbid health conditions, Lin et al. (2010) also found an increased incidence of comorbid conditions linked to chronic stress in patients with OAG compared to those without [25]. Statistically significantly increased incidences of hypertension (*p* < 0.001), ischemic heart disease (*p* < 0.001), hyperlipidemia (*p* < 0.001), congestive heart failure (*p* < 0.001), cardiac arrhythmias (*p* < 0.001), peripheral vascular disorders (*p* < 0.001), diabetes (*p* < 0.001), renal failure (*p* < 0.001), and peptic ulcers (*p* < 0.001) were present in the OAG group compared with controls [25].

In addition to AL, chronic stress related to type A personality has been linked to chronic diseases such as ischemic heart disease. Therefore, a role for type A personality has been suggested in glaucoma [26]. In a study of 50 participants, Bubella et al. (2014) assessed personality type and anxiety in participants with OAG. They discovered that type A personality was evident in 64% of participants with OAG, with higher incidences of state (*p* = 0.03) and trait anxiety (*p* = 0.001) in type A personality. Additionally, type A personality was associated with a higher degree of VF involvement than patients with type B personality (*p* = 0.001) [26]. However, the authors do not clarify the pathophysiological mechanisms behind the observation that subjects with type A personality experience more stress and are at higher risk of VF damage. Therefore, additional research is needed to elucidate the relationship between personality types, chronic stress, and risk of glaucoma onset and progression.

While evidence suggests that patients with chronic stress and elevated AL are more likely to receive glaucoma diagnoses, Salman et al. (2020), found that patients with higher levels of glaucoma-related distress are less likely to adhere to medical treatment guidelines (*p* < 0.001) [27]. Because only one paper focused on this topic, additional, larger studies are needed to further characterize this relationship.

Taken together with the consensus of data above, physical markers for chronic stress such as AL and evidence for long-term chronic stress appear to be associated with increased risk of developing glaucoma and VF damage. Moreover, chronic stress related to personality type appears to also influence the risk of developing glaucoma. Additionally, patients with elevated chronic stress and AL are most at risk for glaucoma diagnoses; however, these same patients are at higher risk for medical treatment noncompliance and, thus, worsening of their condition.

### 4.3. Mental Health

As a result of Engel’s emphasis on psychological health as a factor in the biopsychosocial model of disease, many have investigated the role of mental health conditions on the development of glaucoma. Many have hypothesized a role of psychiatric conditions, including anxiety, depression, and psychosis, on the development of glaucoma. Presently, mixed evidence exists describing the relationship between anxiety and depression on glaucoma [25,28,29,30,31].

Several studies found a significant relationship between psychiatric conditions including anxiety, depression, and psychosis and glaucoma. Lin et al. (2010) conducted a case–control study of OAG and healthy participants and discovered that psychiatric conditions such as depression (*p* < 0.001) and psychosis (*p* < 0.001) were more common in patients with OAG than those without [25]. Additionally, Zhang et al. (2017) found an association between glaucoma and anxiety (OR = 10.6) and glaucoma and depression (OR = 12.3) in a retrospective case–control study of 4,439,518 patients [28]. In another retrospective case–control study, Shin et al. (2021) found a positive association between optic disc hemorrhage (*p* = 0.017), peak IOP (*p* = 0.046), and RNFL thickness loss rate (*p* = 0.019) and high anxiety, as quantified in the Beck Anxiety Inventory [29]. Additionally, high levels of depression, as quantified on the Beck Depression Inventory-II were associated with visual field MD (*p* = 0.006) [29]. Additionally, an Indian study found a correlation between anxiety (*p* < 0.001) and depression (*p* < 0.001), as defined by Hospital Anxiety and Depression (HADS)-Anxiety and HADS-Depression scores, and glaucoma severity [32]. In contrast, Wilson et al. (2022) did not find any statistically significant relationship between glaucoma and depression, defined by either the Epidemiologic Studies Depression Scale or the Composite International Diagnostic Interview Short Form (OR = 0.53) [30]. However, there was a statistically significant relationship between a history of a prior mental health disorder and current depression (OR = 11.26) [30]. Similarly, in a European study, Rezapour et al. (2018) did not find a statistically significant difference in depression (*p* = 0.58) or anxiety (*p* = 0.48) in participants with self-reported glaucoma compared to those without [33]. The relationship between mental health and glaucoma is difficult to investigate and objectively assess, as glaucoma frequently remains undiagnosed [25]. Additionally, patients with existing psychiatric diagnoses may be more likely to seek out medical care, therefore leading to higher rates of glaucoma diagnoses in this population. Biases may also include patient sampling, as research in this field is often limited by racial and ethnically homogeneous populations, thus necessitating larger and more diverse studies [25].

Regarding biological sex among glaucoma patients, women were more likely than men to be depressed (*p* < 0.001), experience stress due to required social distancing from the COVID-19 pandemic (*p* < 0.001), be required to prepare meals alone (*p* = 0.002), or lack help with chores or housekeeping (*p* = 0.003), potentially leading to care delays or worsening of their condition. However, no significant differences existed between having help if unable to leave bed (*p* = 0.09) or having help traveling to the doctor (*p* = 0.75) [34]. However, this study did not investigate the pathophysiology behind the relationship between to biological sex and glaucoma, although, it did investigate the association between the disease and traditional gender roles. Future research should therefore investigate the biochemical or endocrine mechanisms related to biological sex and glaucoma risk.

Alternatively, it was hypothesized that a glaucoma diagnosis could be a catalyst for a depressive event. Wang et al. (2012) also investigated if a glaucoma diagnosis was predictive of depression [31]. In a cross-sectional study of 6760 participants, glaucoma was a predictor for depression when adjusting for age (OR = 1.66, 95% CI 1.09–2.53), sex (OR = 2.15, 95% CI 1.42–3.25), ethnicity (OR = 2.12, 95% CI 1.39–3.23), socioeconomic status (OR = 1.80, 95% CI 1.16–2.79), and comorbidities (OR = 1.59, 95% CI 1.01–2.52). However, after adjusting for general health conditions, glaucoma was no longer a predictor of depression (OR = 1.35, 95% CI 0.822–2.23) [31]. While Wang et al. (2012) utilized a large sample size, only 26.9% of patients who self-reported as glaucomatous also reported use of glaucoma medications [31]. Therefore, the rates of self-reported glaucoma diagnoses may be artificially elevated.

Presently, there is mixed evidence on the role of depression and anxiety in glaucoma. Larger studies evaluating the risk of glaucoma diagnosis and progression in patients with anxiety and depression that carefully account for mitigating factors should be conducted to further understand the dynamic interplay of these conditions. Additionally, larger studies focusing on gender differences in mental health and glaucoma may help further elucidate the dynamic interplay between glaucoma and psychiatric conditions.

### 4.4. Socioeconomic Status

As a chronic disease requiring lifelong treatment, a glaucoma diagnosis burdens patients with high treatment costs worldwide [35]. Additionally, income influences outcomes, with data suggesting there is an inverse association between disease burden and severity, and socioeconomic status worldwide [36]. Moreover, personal- and neighborhood-level poverty appear to be risk factors for developing the condition [35,37].

Treatment for glaucoma, including topical medication and surgical options, is expensive; however, rigid maintenance of therapy is imperative for preservation of vision [35]. A Nigerian study estimates that, on average, glaucoma treatment, transportation, care, and medical testing costs USD 105.40 per month. Included in that sum is an average USD 40 cost for topical glaucoma medications [35]. Other studies estimate that the annual cost of glaucoma treatment in the United States is approximately USD 2.9 billion [38]. Glaucoma represents a leading cause of blindness causing nursing home stay and loss of work, with corresponding costs of USD 11 billion and USD 1.7 billion in the United States [38]. An Italian study demonstrated a linear association between glaucoma severity and cost of treatment [39]. The authors estimated that glaucoma treatment has an approximate cost of EUR 455 to EUR 969 per year, with medications accounting for 42–56% of spending [39].

Additionally, research suggests that poverty, as characterized by not having a personal vehicle to drive to a doctor’s appointment, is associated with an increased risk of glaucoma or suspected glaucoma diagnosis (*p* = 0.001) [40]. Additionally, increased neighborhood poverty, as assessed through the Area Deprivation Index, was associated with an increased risk of glaucoma or suspected glaucoma diagnosis (*p* < 0.0001) [40]. An important limitation of this study is that research participants were recruited from Flint, Michigan, a location with high rates of poverty [40]. Therefore, findings from this study may not be generalizable to other populations. Studies with study participants with different levels of wealth and from different geographic locations are therefore needed to improve the generalizability of the results.

In terms of socioeconomic deprivation, in a Scottish study, Ng et al. (2009) also found a positive association between socioeconomic deprivation, as determined by the Scottish Index of Multiple Deprivation based on home address and glaucoma (POAG or PACG) severity on presentation, defined as loss of VF and presence of paracentral scotoma (*p* = 0.026) [37]. Additionally, in a study of 80 participants with JOAG, lower socioeconomic status was associated with higher odds of VF loss at diagnosis (*p* = 0.01) [41]. However, since this study included data from a tertiary care center, JOAG cases may have presented with worse severity [41]. Moreover, in a prospective cohort study of patients with glaucoma, Salman et al. (2020) found that participants with lower levels of educational attainment and lower socioeconomic status were less likely to adhere to medical therapy guidelines [42]. However, this sample over-represents patients with health insurance (97%), and college degrees (63%) [27]. Additional research is required in a more diverse population.

Glaucoma represents a large economic burden worldwide. Glaucoma treatment requires daily, lifelong treatment to preserve vision. Furthermore, stress from a lack of financial resources is associated with worse status at presentation, worse outcomes, and worse adherence to treatment. Pediatric populations experiencing poverty-induced stress are also at higher risk for worse presentation at diagnosis. Future studies should focus on specific mechanism(s) as targetable interventions to help vulnerable populations.

### 4.5. Racial Stress

Racism and racial stress continue to be a major barrier to access to healthcare for many populations [39]. Additionally, racism itself negatively affects health, in addition to the barriers to access it creates [37]. In relation to glaucoma, evidence suggests that Black patients are more likely to be diagnosed with glaucoma, experience worse outcomes, and have less access to treatment [36,43,44,45].

In terms of glaucoma diagnoses, in a Canadian study, Xie et al. (2024) found that being a non-Hispanic Black patient was associated with a higher likelihood of a glaucoma self-diagnosis (OR = 1.87, *p* < 0.001) [43]. Additionally, Black, Asian, and Hispanic Americans with glaucoma are more likely to present with worse VF MD (*p* < 0.001) than White Americans [44]. Furthermore, Black Americans with glaucoma experience higher VF MD progression per year than White Americans with glaucoma (*p* < 0.001) [44]. Similarly, a Canadian study found that Black glaucoma patients are more likely to present with higher IOP than White glaucoma patients (β = 1.46), even when socioeconomic and genetic factors are accounted for [45].

Despite these findings, Black Americans with glaucoma are less likely to receive outpatient glaucoma treatment (RR = 0.92) and necessary VF testing (RR = 0.92) than White patients with glaucoma [36,45]. However, Black Americans with glaucoma are more likely to receive inpatient glaucoma treatment (RR = 2.42) [36]. Similarly, when compared to White Americans, Hispanic Americans are less likely to receive outpatient glaucoma treatment (RR = 0.97), are less likely to receive necessary RNFL optical coherence tomography (OCT) (RR = 0.87), and are more likely to receive inpatient glaucoma treatment (RR = 2.32) [36]. After stratifying patients by income, when compared to White Americans, Black Americans in the non-low-socioeconomic-status group were still less likely to receive outpatient glaucoma treatment (RR = 0.93), receive VF testing (RR = 0.96), and receive RNFL OCT (R = 0.89), and were more likely to require inpatient hospital glaucoma treatment (R = 2.57) [36].

It is important to highlight that studies investigating the relationship between racial stress and glaucoma risk present several important limitations. First, these studies did not account for participants from multiple racial or ethnic groups; thus, these data are ungeneralizable to diverse populations. Additionally, income level, education, and history of experiencing racial bias are confounding factors in the evaluation of the effect of race on glaucoma diagnosis and progression. Future studies are required to fully tease out the contributions of race, education, income, and personal history experiencing racism as factors influencing glaucoma risk.

The effects of racism and racial stress are still present in medicine and ophthalmology today. When compared to White patients, Black patients with glaucoma are more likely to present with increased severity and progress faster, and are less likely to receive necessary treatment. Additional intervention should focus on early intervention for Black patients at high risk to reduce stress and improve outcomes.

### 4.6. Environment

In addition to socioeconomic status and race, environmental conditions and neighborhood affect glaucoma diagnosis and outcomes. Current evidence suggests that neighborhood factors, including quality and location, affect glaucoma diagnoses, severity, and treatment opportunities worldwide [46,47,48,49].

The neighborhood a person lives in has been shown to significantly impact their quality of life and potential risk for glaucoma. Hicks et al. (2024) demonstrated that neighborhood factors including segregation (*p* < 0.001), neighborhood deprivation (*p* < 0.001), and percentage of the population on Medicaid (*p* < 0.001) are associated with increased glaucoma severity at diagnosis based on VF MD [46]. In pediatric populations with POAG, Elhussainey et al. (2023) found an inverse correlation between neighborhood quality and average IOP (*p* < 0.05), as well as between neighborhood education level and required glaucoma medications (*p* < 0.05) [47]. In pediatric secondary glaucoma patients, higher final visual acuity (VA) is associated with higher neighborhood quality, as measured by the Child Opportunity Index, income, education, and health factors (*p* < 0.001) [47].

Differences in urban and rural settings of neighborhoods may influence glaucoma. Vijaya et al. (2008) investigated prevalence of PACG, Primary Angle Closure (PAC), and Primary Angle Closure Suspect (PACS) in urban and rural South Indian settings as part of the Chennai Glaucoma Study [48]. While the prevalence of PACG and PACS was similar in both populations, PAC prevalence was higher in the urban compared to the rural population (*p* < 0.0001). PAC and PACG were also positively associated with age and IOP in both urban and rural populations and more common in rural women (OR = 4.3; 95% CI, 2.2–8.3). The team also explored the prevalence of POAG in the South Indian population as part of the Chennai Glaucoma Study. The urban population prevalence was significantly greater than the rural population prevalence (1.62%; 95% CI, 1.4%–1.8%; *p* < 0.0001). In both urban and rural populations, IOP and age were significant risk factors associated with disease initiation and progression. They did not find associations with other risk factors such as gender, myopia, hypertension, diabetes, or central corneal thickness [48]. Garudadri et al. (2010) explored the prevalence and risk factors for POAG and PACG in both urban and rural populations from the Andhra Pradesh Eye Disease Study. In POAG (4% vs. 1.6%; *p* < 0.001), PACG (1.8% vs. 0.7%; *p* < 0.01), PAC (0.8% vs. 0.2%; *p* = 0.02), and PACS (3.5% vs. 1.5%; *p* < 0.01), the prevalence was significantly higher in urban communities compared to rural communities [50]. Age and IOP were significant risk factors for POAG in both the urban and rural groups. Blindness owing to POAG was higher in the rural group compared to the urban group (11.1% vs. 2.7%). In PACG, PAC, and PACS, age and IOP were significant risk factors for the urban group, while IOP alone was a significant risk factor in the rural cohort. Female gender was also isolated as a risk factor in the rural cohort (*p* = 0.032). Additional research is required to more clearly understand the differences in risk factors for developing PACG and PACS in urban and rural community [46]. Furthermore, Vijay et al. (2008) and Garudadri et al. (2010) both explored the relationship between environment and glaucoma diagnosis and progression in Indian populations [48,50]. Future research focused on environment in racially, ethnically, and geographically diverse populations will improve overall generalizability and enhance knowledge of the effects of environment on glaucoma.

Time to surgery for glaucoma patients is also affected by their environment. Patients living in the most deprived neighborhoods, as defined by the Area Deprivation Index (ADI) or Social Vulnerability Index, were found to have delayed surgical intervention after controlling for demographic and ocular parameters. Patients from the highest national ADI quartile were associated with a 68% longer time to surgery than glaucoma patients in the least deprived neighborhoods (*p* = 0.002) [49].

Overall data suggest that the quality of neighborhood and environmental conditions (i.e., education and income) have a significant influence on risk factors that drive glaucomatous disease. In most prior research, the prevalence of POAG, PACG, PAC, and PACS is higher in urban communities. Surgical interventions for glaucoma are also delayed in patients from deprived communities with increased social vulnerability.

### 4.7. Mindfulness and Stress Reduction

Stress mitigation techniques may lower the risk for glaucoma. Meditation, mindfulness, relaxation training, and music have demonstrated effectiveness in stress reduction and IOP reduction [51,52,53,54,55]. Nonpharmacologic treatment centered on relaxation and mindfulness may represent a future area of treatment for glaucoma.

Reducing IOP in POAG is currently the gold-standard approach to preventing further optic nerve head damage and glaucoma progression. Kaluza and Strempel (1995) assessed the effect of relaxation training and mental visualization on IOP in POAG patients [51]. Only slight short-term changes in IOP were measured immediately after each training session, but during the training sessions there was a significant decrease in IOP. The 24 h IOP profiles also showed significant reductions in IOP. Medications could also be reduced for 56% of the initially treated patients [51].

In addition to IOP reduction, other mechanisms have been identified as glaucoma drivers, including ischemia, oxidative stress, inflammation/glial activation, and vascular dysregulation. Because stress is a common thread connecting these mechanisms, mindfulness and stress reduction strategies can be useful in lowering IOP and normalizing stress biomarkers [52]. Dada et al. (2018) measured the effects of mindfulness meditation in POAG patients compared to controls. Meditators showed significantly lower IOP compared to controls (OD: 18.8 to 12.7, OS 19.0 to 13.1 mmHg). They also showed significantly reduced stress biomarkers including cortisol (497.3 to 392.3 ng/mL), IL6 (2.8 to 1.5 ng/mL), TNF-α (57.1 to 45.4 pg/mL), reactive oxygen species (1625 to 987 RLU/min/104 neutrophils), elevated β-endorphins (38.4 to 52.7 pg/mL), brain-derived neurotrophic factor (56.1 to 83.9 ng/mL), and total antioxidant capacity (5.9 to 9.3), all *p* < 0.001. Meditators also noted an improved QOL (*p* < 0.05) [52]. While evidence suggests that meditation is associated with reduction in stress biomarkers, it is currently unknown how these biomarkers affect glaucoma risk. Future research is required to understand the dynamic interplay between biochemical markers of stress and glaucoma.

In terms of meditation and mindfulness, Dada et al. (2020) followed their initial work by evaluating mindfulness meditation on IOP and trabecular meshwork gene expression in patients with medically uncontrolled POAG scheduled for trabeculectomy [53]. At 3 weeks, a significant decrease in IOP was seen in the meditation group compared to the control group (21.2 ± 5.6 to 20.0 ± 5.8 mmHg; *p* = 0.38). Moreover, the ΔIOP was significantly greater in the meditation group versus the control group. Gene expression analysis showed significant upregulation of nitric oxide synthetase (NOS1 and NOS3) and neuroprotective genes. Additionally, there was a significant downregulation of proinflammatory genes in the meditation group [53]. In a following study centered on mindfulness, Dada et al. (2021) also examined the effects of mindfulness-based stress reduction (MBSR) on optic disc perfusion in POAG patients with controlled IOP [54]. The MBSR group showed a significant reduction in IOP (*p* = 0.001), an increase in circum-papillary vessel density in the superior (15.8%–17.4%, *p* = 0.02) and nasal quadrants (14.2%–16.5%, *p* = 0.01), and an increase in circum-papillary vascular perfusion in the superior (38.9%–41.1%, *p* < 0.001), temporal (42.2%–44.5%, *p* < 0.001), inferior (40.1%–43.8%, *p* < 0.001), and nasal quadrants (40.6%–42.8%, *p* < 0.001). There was also a significant increase in the Flux Index after 6 weeks (0.38–0.40, *p* < 0.001) [54]. It is important to highlight that the aforementioned studies on mindfulness and medication sampled Indian populations [53,54], thus suggesting the need for larger populations with more diversity in racial and ethnic groups to enhance generalizability.

In addition to mindfulness and relaxation, music therapy may be beneficial in glaucoma management. Bertelmann and Strempel (2015) evaluated music’s short-term effect on both psychological and physiological parameters in glaucoma patients. Best-corrected VA, daily IOP, and short-term mental state development were significantly improved in the music therapy intervention group compared to controls [55]. Wu and Choy (2022) conducted a literature search of psychological interventions such as meditation, autogenic relaxation, music, hypnosis, motivational interviewing, etc., and compared them to patient parameters such as IOP, mental health, and QOL [56]. They found that daily meditation for 30 to 60 min has been shown to improve IOP by 1.5 to 6.1 mmHg, as well as ocular perfusion and QOL. Music, autogenic training, psychological nursing, and bright light therapy have all shown to be beneficial on glaucoma control and vision outcomes, although the results were not significant. Additional research on larger populations is required to more clearly understand the most effectives stress reduction techniques for glaucoma management [56].

Mindfulness meditation and other forms of psychological relaxation have been shown to reduce IOP and stress biomarkers at a genetic level. Psychological relaxation methods may also help bolster the effects of traditional IOP-lowering therapy to control glaucoma progression. Mindfulness and relaxation have also shown significant vascular benefits and reduced vascular damage in patients when IOP is controlled. QOL and mental health have also been shown to be improved when employing psychological relaxation tactics.

## 5. Limitations and Future Directions

An important limitation of this study is the narrative nature of this review. Systematic reviews and meta-analyses on this topic are particularly needed in order to strengthen the results discussed above. Future research efforts should also focus on randomized controlled trials investigating nonpharmacological methods for IOP reduction focused on stress management. While randomized controlled trials are not possible when evaluating socioeconomic status, environment, race, and chronic stress, future retrospective studies may overcome such challenges by including diverse patient populations. Additionally, the current data support IOP elevations associated with acute and chronic stress; however, there is currently far less evidence demonstrating how this IOP increase affects VF outcomes and patients’ quality of life.

## 6. Conclusions

Glaucomatous disease is responsible for vision loss in tens of millions of people worldwide, with elevated IOP as the only modifiable risk factor [1,2]. Stress may significantly impact the risk for developing and experiencing progression of glaucoma. Stress and the biochemical evidence of chronic stress have both been associated with elevated IOP and glaucomatous disease [13,24,25,26]. Acute stress has been shown to increase both IOP and BP, worsen VF, and reduce RNFL thickness [11,16,19,20,21,22,23]. Moreover, AL and chronic stress are linked to worse outcomes for glaucoma patients, possibly due in part to higher rates of medication noncompliance [42]. Furthermore, current research provides mixed evidence for the effects of psychiatric conditions including anxiety and depression on glaucoma, representing an important area for future study in larger populations [25,28,29,30,31].

Social determinants of health, including SES, racism, and environmental conditions, may also impact the diagnosis, treatment, and prognosis of glaucoma. Glaucoma is an expensive condition as it requires lifelong, and often daily treatments [35]. Lower SES is associated with increased frequency of diagnosis and worse severity of the disease [36,37,40]. The effects of racism and racial stress may significantly affect access to healthcare, increasing the likelihood of glaucoma diagnosis and progression [36,39,41,43,44,45,57]. Additionally, neighborhood quality and location affect glaucoma severity and access to treatment [46,47,48,49].

As current treatments for glaucoma are limited to IOP reduction, nonpharmacological stress reduction techniques that reduce IOP are an area of current research [52]. Strategies such as meditation, mindfulness, relaxation, and music all contribute to reductions in sympathetic nervous system activity and reduce IOP, which may therefore improve glaucoma outcomes [51,52,53,54,55]. Future directions in this field should include a multidisciplinary approach to glaucoma management, with collaborations between ophthalmologists and psychiatrists, psychologists, and therapists that could assist with anxiety, depression, and stress reduction techniques [58,59].

Our analysis agrees with previous work [59] summarizing the impact of various forms of stress on glaucoma risk. While our analysis focuses more on acute versus chronic stress, the previous work analyzed the impact of psychiatric conditions, including anxiety and depression, and antidepressant usage and sleep disturbances that may increase the risk for glaucoma. The authors conclude that depression and anxiety were the most common conditions identified, while other conditions, including sleep disorders, psychosis, dementia, and post-traumatic stress disorder, were also observed [59]. Future research should consider acute versus chronic impacts from all forms of stress.

The relationship between glaucoma and stress is an important topic for researchers and clinicians, as it has the potential to enhance the understanding of disease risk in vulnerable populations. Targeted interventions such as glaucoma screening and treatment would be especially beneficial in preserving vision and improving outcomes in individuals exposed to high-stress conditions and individuals experiencing stress due to SES, environment, and racism. Longitudinal research in this field will also improve the understanding of the pathophysiological mechanisms behind stress and glaucoma. Nonpharmacologic stress reduction and relaxation techniques are currently the only available low-cost adjunct to topical pharmacological IOP-lowering mediations. This may help reduce the cost of glaucoma treatment and improve access to care worldwide. Future research in this space is imperative to further the understanding of the implications of stress and stress reduction techniques on glaucoma development and progression to improve vision worldwide. A goal of future research should include longitudinal studies demonstrating the effectiveness of nonpharmacological stress reduction techniques as an adjunct or alternative to topical pharmacologic treatment. An important factor to consider regarding nonpharmacological stress reduction techniques is its scalability in addition to other practical considerations. It may be difficult for mindfulness, meditation, and relaxation to be properly standardized at the current level of treatment. These treatment paradigms would likely occur in the patient’s home, and the levels of adherence and compliance may change from patient to patient. Future research regarding app development and the involvement of AI or telehealth may improve treatment adherence and improve scalability. Additionally, future more in-depth collaboration between ophthalmology and mental health services could improve scalability and inter-operator variability as well [58].

There are major unexplored areas of research investigating therapeutic strategies that address modifiable non-IOP risk factors of glaucoma. Further work is needed to evaluate the risk of glaucoma development and progression in patients with mental health stress due to anxiety and depression, and the varied interactions between these conditions. Additional research should attempt to differentiate the impact of stress on changes to IOP, BP, and RNFL thickness versus medication, limited adherence, and access to quality medical care.

Additionally, while associations have been established between low SES and urban environments on the prevalence of glaucoma, there is a need for further work to derive more robust connections between these societal factors and the genesis of glaucoma in these patients. Future work with larger populations should also consider connections between SES, urban and rural neighborhoods, and the mechanistic linkages to glaucomatous disease.

## Data Availability

No new data were created or analyzed in this study. Data sharing is not applicable to this article.
